# The predictive value of the product of contrast medium volume and urinary albumin/creatinine ratio in contrast-induced acute kidney injury

**DOI:** 10.1080/0886022X.2017.1349673

**Published:** 2017-07-20

**Authors:** Chunrui Wang, Shuai Ma, Bo Deng, Jianxin Lu, Wei Shen, Bo Jin, Haiming Shi, Feng Ding

**Affiliations:** a Division of Nephrology, Shanghai Ninth People's Hospital, School of Medicine, Shanghai Jiaotong University, Shanghai, China;; b Division of Cardiology, Huashan Hospital, Fudan University, Shanghai, China

**Keywords:** Contrast-induced acute kidney injury, coronary angiography, risk factors, urine albumin/creatinine ratio, contrast medium volume

## Abstract

Preexisting renal impairment and the amount of contrast media are the most important risk factors for contrast-induced acute kidney injury (CI-AKI). We aimed to investigate whether the product of contrast medium volume and urinary albumin/creatinine ratio (CMV × UACR) would be a better predictor of CI-AKI in patients undergoing nonemergency coronary interventions. This was a prospective single-center observational study, and 912 consecutive patients who were exposed to contrast media during coronary interventions were investigated prospectively. CI-AKI is defined as a 44.2 μmol/L rise in serum creatinine or a 25% increase, assessed within 48 h after administration of contrast media in the absence of other causes. Fifty patients (5.48%) developed CI-AKI. The urinary albumin/creatinine ratio (UACR) (OR = 1.002, 95% CI = 1.000–1.003, *p* = .012) and contrast medium volume (CMV) (OR = 1.008, 95% CI = 1.001–1.014, *p* = .017) were independent risk factors for the development of CI-AKI. The area under the ROC curve of CMV, UACR and CMV × UACR were 0.662 (95% CI = 0.584–0.741, *p* < .001), 0.761 (95% CI = 0.674–0.847, *p* < .001) and 0.808 (95% CI = 0.747–0.896, *p* < .001), respectively. The cutoff value of CMV × UACR to predict CI-AKI was 1186.2, with 80.0% sensitivity and 62.2% specificity. The product of CMV and UACR (CMV × UACR) might be a predictor of CI-AKI in patients undergoing nonemergency coronary interventions, which was superior to CMV or UACR alone.

## Introduction

Contrast-induced acute kidney injury (CI-AKI) is defined as an abrupt deterioration in renal function associated with the administration of iodinated contrast media, and it is the third most common cause of hospital-acquired acute kidney injury (AKI) after impaired renal perfusion and use of nephrotoxic medications [1–3]. Among all procedures utilizing contrast media for diagnostic or therapeutic purposes, coronary angiography (CAG) and percutaneous coronary intervention (PCI) are associated with the highest rates of CI-AKI [3,4]. Although it is still not completely understood, medullary hypoxia and direct tubular toxicity of the contrast media are accepted as the main pathophysiological mechanisms of CI-AKI [5,6]. CI-AKI can result in prolonged hospital stay, occasional need for dialysis, permanent decrease in residual renal function and increased mortality [7,8]. Because there is no effective therapy once kidney injury has occurred, accurate and timely identification of patients at risk may have a substantial impact on it [9]. Although many risk factors have been described for CI-AKI, preexisting renal impairment is the most important one, and the amount of contrast media is the main modifiable one [4,10]. Previous studies have shown that the ratio of contrast medium volume to creatinine clearance (CMV/CrCl) [11,12] and the ratio of contrast medium volume to estimated glomerular filtration rate (CMV/eGFR) [13,14] may be predictors of CI-AKI after PCI. However, there is an increasing recognition that measurement of serum creatinine does not provide an ideal estimation of renal function, and even equations which estimate kidney function with corrections for age, weight and gender, such as the Modification of Diet in Renal Disease (MDRD) and Cockcroft–Gault have limitations [15]. Albuminuria is a direct consequence of renal glomerular/tubular injury and increases with glomerular dysfunction [16]. And it is a known marker for progression of chronic renal disease. However, it also responds to changes in diet, exercise, clinical conditions (such as fever) and medications. Spot urine albumin/creatinine ratio (UACR) is a reasonable surrogate for 24-h urine albumin excretion rate and certainly not without limitations [15]. We hypothesized that the product of CMV and UACR (CMV × UACR) would be a better predictor than CMV or renal function alone in patients undergoing coronary interventions, including CAG and PCI.

## Methods

### Study population

This was a prospective single-center observational study, conducted in a tertiary hospital, from March 2010 to February 2011, included consecutive patients underwent nonemergency coronary interventions (CAG and PCI). The hospital ethics committee of Shanghai Ninth People’ Hospital, School of Medicine, Shanghai Jiaotong University proved the research protocol (approval number: 2009-206), and all included patients gave their written informed consent. The inclusion criteria were patient age ≥18 years, measurement of serum creatinine within 7 days before coronary interventions and within 48 h after the procedure. The exclusion criteria were patients aged <18 years and patients who were admitted to the emergency department or the intensive care unit. Patients were also excluded from the study if the serum creatinine ≥707 μmol/L or fluctuated more than 44.2 μmol/L 24 h before coronary interventions; requiring maintenance hemodialysis or peritoneal dialysis; having a history of blood system diseases, liver disease or cancer; being pregnant or lactating women; exposed to contrast media within 7 days or allergic to contrast media; not wishing to participate or their data were incomplete. The study was also registered at www.clinicaltrials.gov (NCT01142024).

### Study protocol

We recorded the demographic information of each patient including age, gender, body mass index (BMI), comorbid diseases, and current medication used by them. Results of laboratory blood biochemical tests and echocardiogram before the coronary intervention, type and volume of contrast media during the procedure were documented. Serum creatinine concentrations were routinely measured at the time of admission, 24 h and 48 h after the procedure. CI-AKI is defined as a 44.2 μmol/L rise in serum creatinine or a 25% increase, assessed within 48 h after administration of contrast medium in the absence of other causes [4]. Chronic renal failure was defined as a glomerular filtration rate (GFR) < 60 mL/min/1.73m^2^, estimated with the modified MDRD formula [17]. All patients were given intravenous hydration with isotonic (0.9%) saline 500 mL, 1 mL/kg/h, at the beginning of coronary interventions. In patients with heart failure (left ventricular ejection fraction lower than 40%), the infusion rate was reduced to 0.5 mL/kg/h. General patients received nonionic monomer, low-osmolar contrast agents (iopamidol). As the hospital CI-AKI prevention protocol, the contrast agents were changed to iodixanol, a nonionic dimer, iso-osmolar media, in patients with high-risk factors (such as age >75 years, anemia, diabetes, preexisting kidney disease and heart failure).

### Statistical analysis

Continuous variables were expressed as mean ± SD or median with range of quartile, and categorical variables were expressed as percentage. In univariate analysis, comparisons between groups were analyzed using independent *t*-test or Mann–Whitney U-test for continuous variables and Pearson chi test or Fisher exact test for categorical variables. Binary logistic regression analysis was used for multivariate analysis. The odd ratios (ORs) and 95% confidence intervals (CIs) were calculated. The receiver operating characteristics (ROC) curve was used to calculate the sensitivity and specificity of the principal variable level, and optimal cutoff value (Youden Index) for predicting CI-AKI following nonemergency coronary interventions. All statistical analyses were performed with SPSS 17.0 (Chicago, IL), and a two-sided *p* < .05 was considered significant.

## Results

### Patient characteristics

From March 2010 to February 2011, all consecutive patients (*n* = 949) who underwent nonemergency coronary interventions (CAG and PCI) admitted to our hospital were considered for enrollment in the study. Patients who did not wish to participate (*n* = 24) were exposed to contrast media within 7 days (*n* = 5), were requiring maintenance hemodialysis (*n* = 4) and with the absence of data on serum creatinine during the 48 h following the procedure (*n* = 4) were excluded. The remaining 912 eligible patients were enrolled. A total of 50 patients (5.48%) developed CI-AKI. The mean age of the patients (589 men and 323 women) enrolled in the study was 66.1 ± 10.6 years. Twenty-nine patients (3.2%) received iodixanol, and 883 patients (96.8%) received iopamidol. The mean volume of contrast media (CMV) was 132.1 ± 79.5 mL. Comorbid diseases and current medications were shown in Table 1.

**Table 1.** Baseline clinical and procedural-related characteristics of patients with and without CI-AKI.

	CI-AKI	no CI-AKI	
	(*n* = 50, 5.48%)	(*n* = 862, 94.52%)	*p*
Age, years	68.6 ± 10.3	66.0 ± 10.6	.073
Male, *n* (%)	27 (54.0)	562 (65.2)	.108
BMI, kg/m^2^	24.6 ± 3.4	24.6 ± 3.8	.534
PCI, *n* (%)	32 (64.0)	337 (39.1)	<.001
Iodixanol, *n* (%)	3 (6.0)	26 (3.0)	.243
CMV, mL	204.8 ± 93.1	127.3 ± 76.4	<.001
Comorbid diseases, *n* (%)			
Hypertension	38 (76.0)	590 (68.4)	.262
Diabetes mellitus	20 (40.0)	205 (23.8)	.010
Heart failure	3 (6.0)	35 (4.1)	.505
Renal failure	9 (18.0)	97 (11.3)	.148
Current medications, *n* (%)			
ACEIs/ARBs	29 (58.0)	447 (51.9)	.542
Biguanides	8 (16.0)	124 (14.4)	.148
Diuretics	6 (12.0)	101 (11.7)	.455

ACEIs: angiotensin-converting enzyme inhibitors; ARBs: angiotensin receptor blockers; BMI: body mass index; CI-AKI: contrast-induced acute kidney injury; CMV: volume of contrast media; PCI: percutaneous coronary intervention.

Univariate analysis (Tables 1 and 2) had identified several risk factors for CI-AKI. The level of serum NT-proBNP, UACR and CMV was significantly higher in CI-AKI patients. There were much more patients with diabetes mellitus in CI-AKI group (40.0% vs. 23.8%, *p* = .010). And the hemoglobin (*p* = .001) and serum prealbumin (*p* = .008) levels on admission were lower in patients with CI-AKI than in those without. In CI-AKI group, more patients underwent PCI (64.0% vs. 39.1%, *p* < .001). Of note, neither eGFR (*p* = .843) nor renal failure (*p* = .148) was a significant risk factor in our population.

**Table 2.** Laboratory characteristics of patients with and without CI-AKI.

	CI-AKI	no CI-AKI	
	(*n* = 50, 5.48%)	(*n* = 862, 94.52%)	*p*
Hb (g/L)	125.2 ± 21.4	135.1 ± 15.9	.001
FBG (mmol/L)	5.1 ± 1.7	5.7 ± 1.6	.526
2h-PBG (mmol/L)	7.6 ± 2.2	7.9 ± 2.9	.861
HbA1c (%)	6.7 ± 1.4	6.3 ± 1.1	.108
TG (mmol/L)	1.9 ± 1.4	1.8 ± 1.2	.521
TC (mmol/L)	4.5 ± 1.4	4.3 ± 1.1	.353
LDL (mmol/L)	2.6 ± 0.9	2.5 ± 0.8	.269
HDL (mmol/L)	1.0 ± 0.3	1.0 ± 0.3	.186
NT-proBNP (pg/L)	325.5 (92.7, 2962.5)	133.5 (47.4, 453.9)	<.001
LVEF (%)	61.3 ± 13.0	64.6 ± 9.6	.124
Prealbumin (g/L)	197.7 ± 47.6	214.5 ± 49.3	.008
hs-CRP (mmol/L)	1.3 (0.5, 5.2)	1.3 (0.5, 3.8)	.566
Scr (μmol/L)	69.0 (58.5, 97.0)	75.0 (64.0, 86.0)	.337
BUN (mmol/L)	7.0 ± 3.3	6.1 ± 1.8	.059
eGFR (mL/min/1.73m^2^)	87.8 ± 40.1	86.1 ± 22.8	.843
UACR (mg/mmol)	26.5 (6.3, 253.9)	7.2 (4.7, 15.4)	<.001

BUN: blood urea nitrogen; eGFR: estimated glomerular filtration rate; FBG: fasting blood glucose; Hb: hemoglobin; HbA1c: hemoglobin A1c; HDL: high-density lipoprotein; hs-CRP: high-sensitivity C-reactive protein; LDL: low-density lipoprotein; LVEF: left ventricular ejection fraction; NT-proBNP: NT-pro-brain-natriuretic peptide; 2 h-PBG: 2-h postprandial glucose; Scr: serum creatinine; TC: total cholesterol; TG: triglyceride; UACR, urine albumin/creatinine ratio.

### UACR and CMV of patients with and without CI-AKI

The factors that were significant in univariate analysis and other classic predictors (age, gender, eGFR) were then studied in multivariate analysis using forward logistic regression to discover independent predictors (Table 3). UACR (OR = 1.002, 95% CI = 1.000–1.003, *p* = 0.012) and CMV (OR = 1.008, 95% CI = 1.001–1.014, *p* = .017) remained significant independent correlates of CI-AKI. Logistic equation was shown below:

p=0.008 × CMV + 0.002 × UACR - 0.002

**Table 3.** Multivariate analyses of UACR, CMV and other known predictors of CI-AKI.

	OR	95% CI	*p*
Gender	0.805	0.206, 3.136	.754
Age	1.105	0.236, 5.167	.899
DM	1.437	0.781, 2.645	.244
Prealbumin	0.998	0.984, 1.012	.805
Hb	0.994	0.956, 1.033	.765
NT-proBNP	1.000	0.999, 1.000	.378
eGFR	1.030	1.000, 1.061	.510
PCI	0.543	0.115, 2.563	.440
CMV	1.008	1.001, 1.014	.017
UACR	1.002	1.000, 1.003	.012

CMV: volume of contrast media; DM: diabetes mellitus; eGFR: estimated glomerular filtration rate; Hb: hemoglobin; NT-proBNP: NT-pro-brain-natriuretic peptide; PCI: percutaneous coronary intervention; UACR: urine albumin/creatinine ratio.

Hosmer–Lemeshow goodness-of-fit test showed that the model fitting was good (*p* = .771).

**Figure 1.**
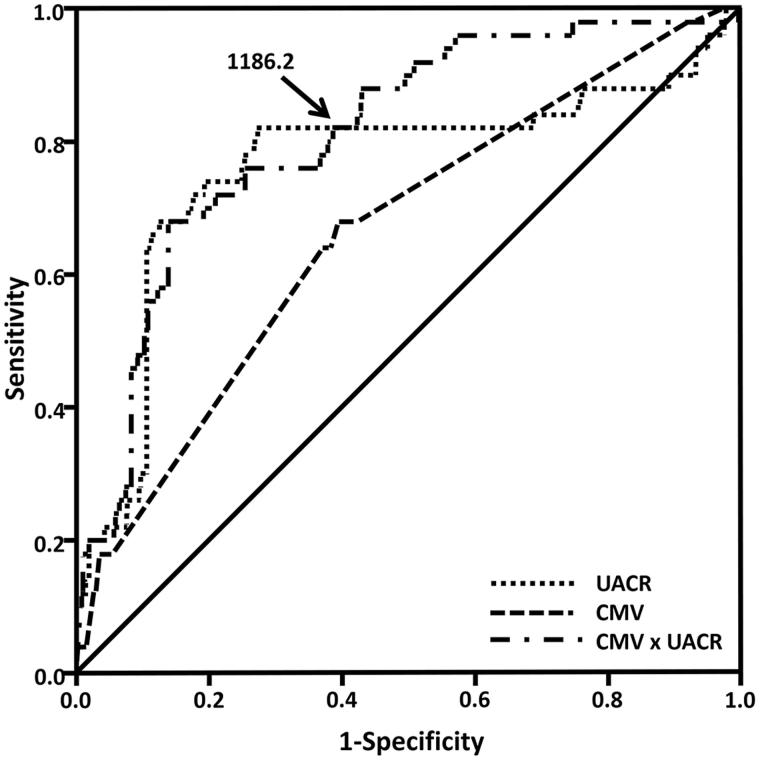
The receiver operating characteristic curve of CMV, UACR and the product of CMV and UACR (CMV × UACR). The cutoff value (Youden index) of CMV × UACR to predict CI-AKI was 1186.2, with 80.0% sensitivity and 62.2% specificity. Abbreviations: CMV, volume of contrast media; UACR, urine albumin/creatinine ratio; CMV × UACR, the product of CMV and UACR.

**Figure 2.**
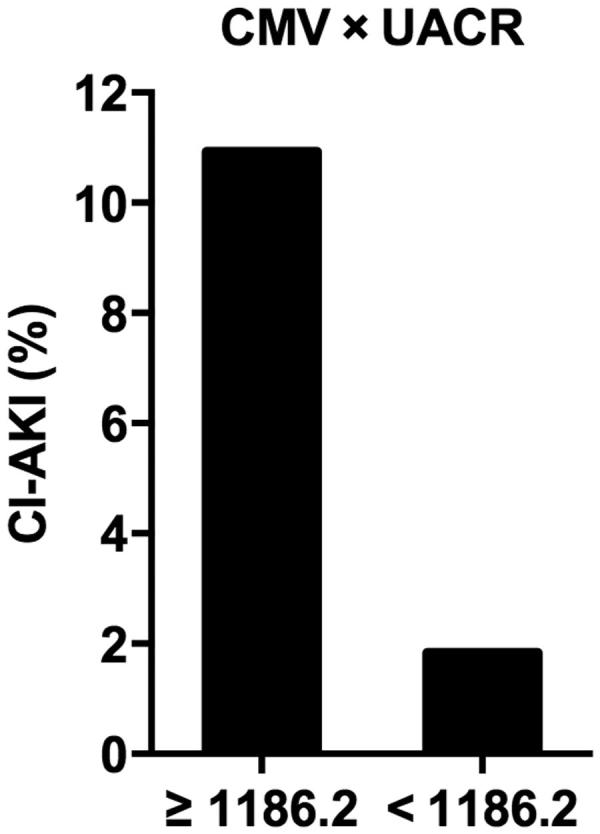
The CI-AKI incidence and CMV × UACR. *p* < .001. Abbreviations: CMV, volume of contrast media; UACR, urine albumin/creatinine ratio; CMV × UACR, the product of CMV and UACR.

Finally, the ROC curve analysis was performed to determine the cutoff value (Youden Index) of UACR and CMV to predict CI-AKI (Figure 1). The area under the ROC curve of CMV, UACR and the product of CMV and UACR (CMV × UACR) were 0.662 (95% CI = 0.584–0.741, *p* < .001), 0.761 (95% CI = 0.674–0.847, *p* < .001) and 0.808 (95% CI = 0.747–0.896, *p* < .001), respectively. The cutoff value of CMV × UACR to predict CI-AKI was 1186.2, with 80.0% sensitivity and 62.2% specificity. In patients with CMV × UACR ≥1186.2, the incidence of CI-AKI was 10.93% (40/366), compared with 1.83% (10/536) in patients with CMV × UACR <1186.2 (*p* < .001, Figure 2).

## Discussion

The incidence of CI-AKI in our monocentric population undergoing nonemergency coronary interventions (CAG and PCI) was 5.48%. This proportion is similar to the data available in the literature [5,10]. The two-day mortality rate in CI-AKI patients was 0% in our study, and it showed no need for renal replacement therapy. CI-AKI generally occurs within 48 h of contrast exposure, the increase in serum creatinine peaking 5–7 days later [4,18,19]. Because almost all of our patients were discharged 2 days after coronary interventions, few data were available regarding longer-term follow-up, the incidence of CI-AKI and long-term effects might be underestimated.

Our study found that CMV was one of the independent risk factors for the development of CI-AKI. Many studies have confirmed that the large dose of contrast media is one of the risk factors for CI-AKI. In 1989, Cigarroa et al. described how adherence to a formula for a contrast media limit could be used to significantly reduce the rates of CI-AKI. Cigarroa correctly predicted that the incidence of CI-AKI would be related to the dose of contrast media and inversely proportional to creatinine. Their formula was: contrast media limit = 5 mL of contrast per kilogram body weight/Scr (mg/dL), maximum dose of 300 mL [20]. In a retrospective analysis of 16,592 patients undergoing PCI found that the exceeding Cigarroa’s limit was the strongest predictor with an OR of 6.2 (95% CI = 3.0–12.8) [21]. The pathogenesis of CI-AKI is still not completely understood, at least two significant processes are known to be involved in the pathophysiology of CI-AKI, vasoconstriction resulting in medullary hypoxia and direct toxicity caused by the contrast media to renal tubular cells. The mechanisms that have been implicated in these processes are dehydration, decreased prostaglandin and nitric oxide induced vasodilatation, impaired endothelial function, increase in renal adenosine concentration, increase in oxygen free radicals in response to hyperosmotic load, increased intratubular pressure owing to contrast-induced diuresis, increased urinary viscosity and obstruction of the tubules [22,23]. Because the contrast media dosage are considered modifiable, in the process of coronary interventions, to limit the use of contrast media and avoid repeated angiography are important. Our study showed that more patients underwent PCI in CI-AKI group, due to the exposure to larger dose of contrast media during the procedure.

Although many risk factors have been described for CI-AKI, preexisting renal impairment is the one of the most important. In our population, neither eGFR nor renal failure emerged as a predictor. The eGFR was almost normal, and the percentage of patients with renal failure was low. There was a trend for a higher rate of renal failure in CI-AKI patients, but the small number of patients was certainly the major reason why this classic risk factor did not emerge from our study. Also our population was undergoing nonemergency coronary interventions, who were at low risk compared to emergency patients. And there is an increasing recognition that measurement of serum creatinine does not provide an ideal estimation of renal function, and even equations that estimate kidney function with corrections for age, weight and gender. In the early stage of renal injury, eGFR is not decreased significantly, but microalbuminuria often can be detected [24].

In our study, UACR was significantly higher in CI-AKI patients, which was an independent risk factor of CI-AKI. Albuminuria is a direct consequence of renal glomerular/tubular injury and increases with glomerular dysfunction. It is also a known marker for the progression of chronic renal disease [15,16]. The association between proteinuria and CI-AKI can be related to their toxic effects on tubular system [8]. There are several data assuming contrast media and proteinuria share similar effects on tubular system injury including the activation of Fas-mediated and peroxisome proliferator-activated receptor-γ-dependent apoptosis and the induction of proinflammatory molecules [8]. But albuminuria also responds to changes in diet, exercise, clinical conditions (such as fever) and medications. Spot urine albumin/creatinine ratio (UACR) is a reasonable surrogate for 24-h urine albumin excretion rate, and certainly not without limitations [15].

We found that using CMV × UACR to predict CI-AKI was superior to using CMV or UACR alone. In patients with CMV × UACR ≥1186.2, the incidence of CI-AKI was 10.93% (40/366). The CMV × UACR is easy to calculate and therefore is a clinically feasible method for determining the maximum safe contrast volume during nonemergency coronary interventions. Laskey et al observed that the CMV/CrCl >3.7 was an independent predictor for an early abnormal increase in Scr after PCI [12]. The predictive value of the CMV/CrCl for CI-AKI after PCI was also confirmed in high-risk patients with cardiac systolic dysfunction [25]. Similarly, previous studies have shown that the CMV/eGFR were predictors of CI-AKI after PCI [13,14]. However, these previous studies were focused on high-risk patients. So in our low-risk population, neither CrCl nor eGFR was a best choice to estimate preexisting renal impairment. Therefore, for patients with increased UACR, in order to reduce the incidence of CI-AKI patients, trying to reduce CMV is important.

### Study limitations

This was a prospective, observational study, conducted at a single medical center. The number of patients undergoing nonemergency coronary interventions (CAG and PCI) was small, which may make this study under powered. As an observational study, lack of a validation cohort, the causality between elevated CMV × UACR and the development of CI-AKI cannot be confirmed. In addition, long-term effects of CIN were not assessed. Finally, as the study population were at low risk, the cutoff value of the CMV × UACR might be changed in other populations.

## Conclusions

The product of CMV and UACR (CMV × UACR) could be a useful predictor of CI-AKI in patients undergoing nonemergency coronary interventions, which was superior to CMV or UACR alone, especially in patients with normal baseline level of Scr and eGFR. When CMV × UACR≥ 1186.2, the incidence of CI-AKI was much higher.
